# Supplementary material for: Cycles of Andean mountain building archived in the Amazon Fan
Source: Nat Commun. 2022 Nov 15;13:6983. doi: 10.1038/s41467-022-34561-6 (PMC9666610; doi:10.1038/s41467-022-34561-6)
Supplement: Supplementary file 1 — Supplementary Information [file 41467_2022_34561_MOESM1_ESM.pdf]

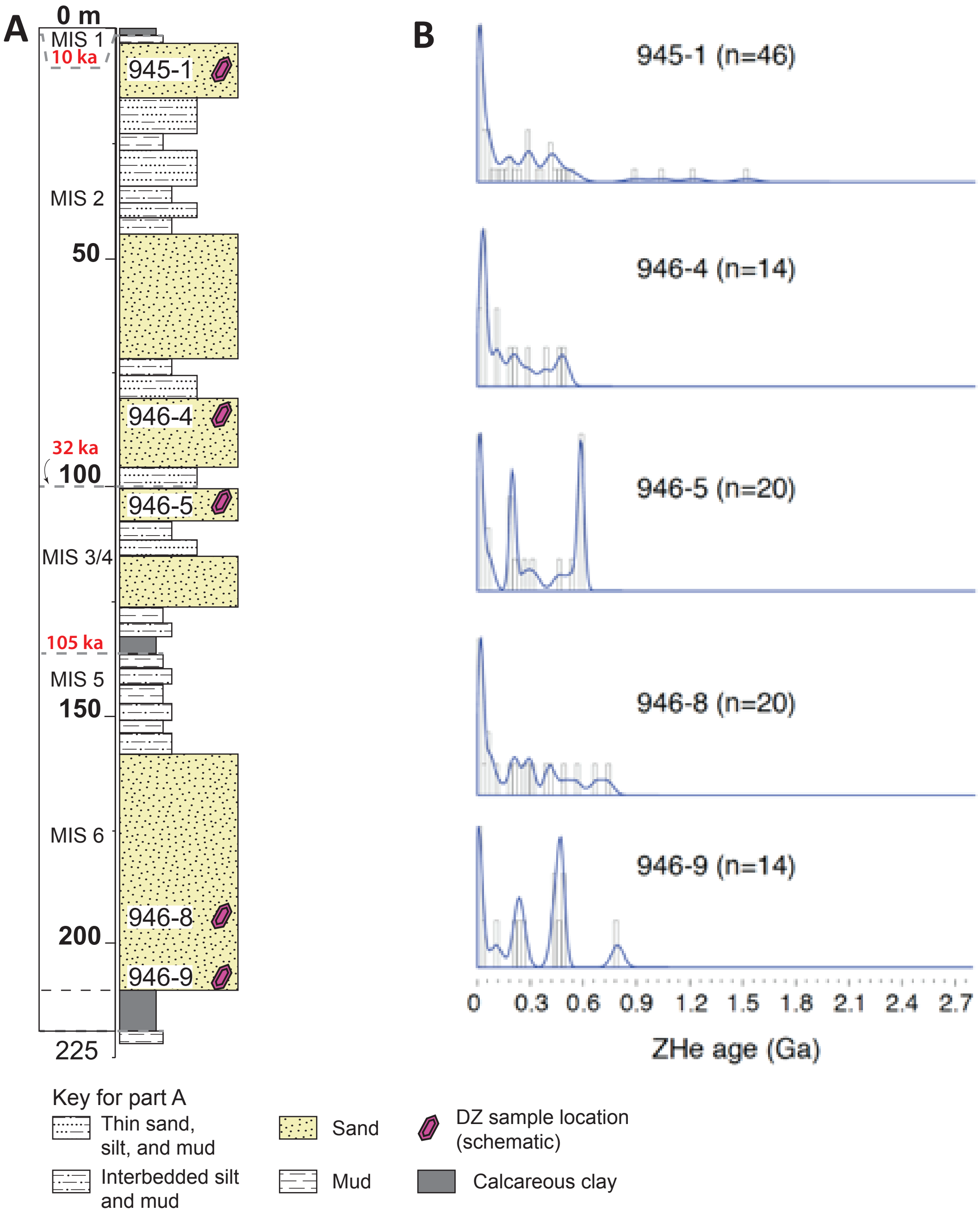

Supplementary Figure: A: Composite lithostratigraphic section for ODP Leg 155, sites 945 and 946. B: DZ U-Th/He ages for each sub-sampled interval.
