# Supplementary material for: Cycles of Andean mountain building archived in the Amazon Fan
Source: Nat Commun. 2022 Nov 15;13:6983. doi: 10.1038/s41467-022-34561-6 (PMC9666610; doi:10.1038/s41467-022-34561-6)
Supplement: Supplementary file 3 — Description of Additional Supplementary Files [file 41467_2022_34561_MOESM3_ESM.pdf]

Description of Additional Supplementary Files

File Name: Supplementary Data 1
Description: U-Th/He isotopic measurements used in double dating of detrital zircons. Complete U-Pb isotopic measurements are available from Mason et al. (2019; Geology) as a supplementary data file.
